# Determinants of patient enablement at primary health care centres in central Ethiopia: a cross-sectional study

**DOI:** 10.4102/phcfm.v3i1.195

**Published:** 2011-04-18

**Authors:** Zewdie Birhanu, Mirkuzie K. Woldie, Tsion Assefa, Sudhakar Morankar

**Affiliations:** 1 Department of Health Education and Behavioural Sciences, Jimma University, Ethiopia; 2 Department of Health Services Management, Jimma University, Ethiopia

## Abstract

**Background:**

Patient enablement is associated with behaviours like treatment adherence and self-care and is becoming a well-accepted indicator of quality of care. However, the concept of patient enablement has never been subjected to scientific inquiry in Ethiopia.

**Objectives:**

The aim of this study was to determine the degree of patient enablement and its predictors after consultation at primary health care centres in central Ethiopia.

**Method:**

Data were collected from 768 outpatients from six primary health care centres in central Ethiopia during a cross-sectional study designed to assess patient satisfaction. Consecutive patients, 15 years or older, were selected for the study from each health centre. Multinomial logistic regression was performed to identify predictors of patient enablement using SPSS (version 16.0).

**Results:**

The study showed that 48.4% of patients expressed an intermediate level of enablement, while 25.4% and 26.2% of the patients expressed low and high levels of patient enablement, respectively. Four models were developed to identify predictors of patient enablement. The first model included socio-demographic variables, showing that residence, educational status and occupational status were significantly associated with patient enablement (*p* < 0.05). This model explained only 20.5% of the variation. The second and third models included institutional aspects, and perceived doctor–patient interaction and information sharing about illness, respectively. They explained 31.1% and 64.9% of the variation. The fourth model included variables that were significantly associated with patient enablement in the first, second and third models and explained 72% of the variation. In this model, perceived empathy and technical competency, non-verbal communication, familiarity with the provider, information sharing about illness and arrangement for follow-up visits were strong predictors of patient enablement (*p* < 0.05).

**Conclusion:**

The present study revealed specific predictors of patient enablement, which health care providers should consider in their practice to enhance patient enablement after consultation.

## Introduction

The consultation – the encounter between health care provider and patient – is the core activity of clinical medicine. As such, the consultation has rightly attracted a good deal of attention, particularly in the primary health care setting, where the vast majority of doctor–patient encounters take place.^1, 2^ Quality of care integrates the notions of access to care and interpersonal effectiveness.^3^ Interpersonal effectiveness is widely regarded as one of the core attributes of good primary care practice.^4^ With interpersonal effectiveness as focus, the concept of patient enablement reflects the extent to which patients understand their health problems and feel able to cope with them as a result of the consultation. It describes the effect of the clinical encounter on patients’ ability to cope with and understand their illness, incorporates the notion of encouragement and enables patients to realise their autonomy and empowerment.^5^ Patients find it very important to be able to understand the nature of their problem and manage their own illness,^6^ which supports the concept of enablement as a patient-specific health-related benefit resulting from a consultation. Patient enablement is based on the principles of patient-centred care and holism.^7^

Enablement is an indicator of the self-efficacy benefits of consulting a health care provider and is expected to be associated with behaviours like treatment adherence and self-care and indicators of quality of care.^5^ Studies revealed that provider empathy plays a significant role in determining the outcome of consultation enablement and is often seen as crucial to achieving patient centredness.^8^ Empathy enhances the provider–patient relationship and therapeutic efficacy, decreases patient anxiety (which itself is linked to physiologic effects), improves patient enablement and has shown clear links to patient health outcome.^9–11^ Non-verbal communication is also believed to be an important factor contributing to patient enablement. Providers who appear fully attentive, avoid distractions, smile and sit on the same level as the patient create an impression of caring, listening and expressing empathy. Such non-verbal cues and language often help to put patients at ease and enhance the patient enablement. In addition, a calm, clean and well-functioning environment that is comfortable and inviting communicates a respect for and commitment to patients and their needs,^12^ but are often ignored in medical research and practice.

Ethiopia has extremely poor health status compared to other low-income countries. Patient empowerment is one the strategies to reduce the burden of communicable diseases, is crucial in the management of clinical cases and plays a significant role in the effort to attain the Millenium Development Goals. With this in mind, the Ethiopian government has committed to deliver quality health care to the population, which reflects in health policy and health sector programmes. Patient enablement is a reliable indicator of the quality of care. Knowing the extent to which the patient feels enabled as a result of consultation plays a significant role in the strategy and tactics a health care provider uses in delivering services to clients.^13–15^ The quality of care can therefore not be considered without thinking about the quality of consultation, which is the central concern of clients. However, patient enablement following a consultation has not yet been subjected to scientific investigation, particularly in Ethiopia. To facilitate implementation of government policy and commitment to the initiative to provide quality care to the population, it is a timely and appropriate to assess the extent to which patients feel enabled and empowered following a consultation. This can, in turn, serve as an indication of the quality of the consultation and, hence, this study was aimed at determining the level of patient enablement and its determinants in primary health care consultation.

## Ethical considerations

This study was approved by the Ethical Clearance Committee of the Jimma University. Verbal informed consent was sought from all the respondents before the start of each interview.

## Methods

### Study design and participants

The data were collected as part of a cross-sectional study conducted in West Shoa, central Ethiopia between December 2008 and January 2009. West Shoa is one of the 17 zones of the Oromia Regional State in Ethiopia and comprises 21 districts. The zone has an estimated total population of 2 072 485 of whom 1 037 159 are females.

The study population consisted of patients who visited the adult outpatient departments of six health centres in West Shoa during the study period. Only patients aged 15 years or older were included in the study, which resulted in 768 patients participating in the survey. All cases of the patient satisfaction survey were included in the analysis. One urban and five rural health centres were randomly selected. The sample size from each centre was determined proportionally according to the number of suitable patients who visited the outpatients department during the 10 days before data collection commenced. Finally, consecutive patients who fulfilled the inclusion criterion were included in the study until the allocated size was obtained for all six health centres. A detailed description of the method can be found elsewhere.^16^

### Measurements

The following instruments were adapted from similar studies.

#### Consultation and Relational Empathy (CARE)

The Consultation and Relational Empathy (CARE) instrument measures patients’ perception of providers’ empathy during the clinical encounter. Patients have to rate aspects of empathy in 10 questions, with each question being scored on an ordinal scale from 1 (poor) to 5 (excellent). Scores are then added, with the maximum possible score being 50 and the minimum 10. Perceived empathy was categorised as indicating low (0–24), medium (25–37) and high enablement (38–50).

The 10 items asked patients to rate their health care provider on (1) the ability to make them feel at ease, (2) allowing them to tell their 'story’, (3) level of listening, (4) the amount of interest shown in the patient as a person, (5) fully understanding patient concerns, (6) level of care and compassion, (7) positive outlook, (8) manner of explanation, (9) helping patients take control, and (10) involving patients in decisions about their treatment plan.

The Cronbach alpha co-efficient was 0.964, which indicated good internal consistency for the empathy scale.

#### Perceived technical competence

Patients’ perception of their providers’ technical competence relates to their subjective judgement of professional skills and providers’ ability to make a diagnosis. It was measured according to eight items, each scored on a 5-point Likert scale ranging from 1 (strongly disagree) to 5 (strongly agree). Scores could range between 8 and 40. Perceived technical competency was categorised as indicating low (0–19), medium (20–30) and high enablement (31–40). The scale had high internal consistency (Cronbach's *α* = 0.910). The items addressed physical examinations, procedural steps to arrive at a diagnosis, the providers’ level of experience, etc.

#### Perceived non-verbal communication of the provider

Non-verbal communication refers to providers’ communication without linguistic content. It was measured according to eight items on a 5-point Likert scale that ranged from 1 (poor) to 5 (excellent). The items addressed aspects of non-verbal communication such as eye contact, forward leaning, posture, facial expression, head nods, hand gestures, emotional expressions and tone of voice. Reliability analysis showed the scale to have high internal consistency (Cronbach's *α* = 0.935). Perceived non-verbal communication scores were categorised as indicating low (0–20), medium (21–29) and high enablement (30–40).

#### Consultation length

Consultation length refers to the amount of time patients spend with the health care provider in the consultation room. A watch was used to record the amount of time patients spent with the health care providers.

#### Information sharing about illness

The extent to which patients were given relevant information related to their illness was assessed according to five items. These included (1) being told the name of their illness and (2) its cause, (3) being given advice to prevent re-occurrence or (4) future development of a similar condition, and (5) being told to return. These items were answered with yes/no responses. In addition to the above dimension and instruments, questions related to institutional aspects and visiting patterns were included.

#### Patient enablement

Patient enablement is the immediate effect of clinical encounters on patients’ ability to cope with and understand their illnesses and indicates quality of consultation.^17, 18^ It was measured with a standardised patient enablement instrument, which addresses six questions regarding a patient's recent visit. These addressed whether they felt able to (1) cope with life, (2) understand their illness, (3) cope with their illness, (4) keep healthy, (5) felt confident about their health, and (6) able to help themselves. All items were stated positively and responses were scored on an ordinal scale (same or less = 0; better or more = 1; much better or much more = 2). Responses were added together for scores ranging between 0 and 12. The scale was found to be reliable (Cronbach's *α* = 0.897). Scores were categorised to indicate low (0–4), medium (score 5–9), and high enablement (10–12).

The questionnaires were translated into Afan Oromo and translated back to English, and subsequently checked for translation consistency by several people. The Afan Oromo version was pre-tested on a sample from a similar population, 5% the size of the total sample. Data were collected by trained individuals.

### Statistical analysis

The data were analysed using statistical software (SPSS 16.0). The frequency distributions of all variables were examined to check for data entry errors. Multinomial logistic regression was performed to identify independent predictors of patient enablement. Four models were developed as part of the analysis to examine the effect of different categories of explanatory variables on the dependent variable. The first model assessed the effects of socio-demographic variables, the second the effects of institutional variables, and in the third the interaction-related variables were included. From the three models, explanatory variables which had statistically significant association with the outcome variable (*p* < 0.05) were entered into the final multinomial logistic regression model based on a likelihood ratio. A 95% confidence interval (CI) and significance level set at less than 0.05 were used to evaluate association between independent and dependent variables.

## Results

### Socio-demographic determinants of patient enablement

Table 1 presents socio-demographic determinants of patient enablement. About half (48.4%) of the patients felt that they experienced an intermediate level of enablement, while 25.4% and 26.2% experienced low and high levels of enablement, respectively. Residence, educational status and occupational status were found to be significantly associated with patient enablement (*p* < 0.05). With intermediate enablement as reference, the adjusted odds ratio (AOR) showed that urban respondents were 0.40 times less likely to experience low enablement than those from rural areas (AOR = 0.40, 95% CI = 0.25–0.65, *p* = 0.001). Conversely, respondents from urban areas were 1.70 times more likely to experience high enablement than their rural counterparts (AOR = 1.70, 95% CI = 1.10–2.63, *p* = 0.016). The pseudo R-square value showed that this model explained 20.5% of the variation.

**TABLE 1:** Socio-demographic determinants of patient enablement at primary health care centres in central Ethiopia.

Explanatory variables	Level of enablement (predicted)			

	Low		High	
	
	*p*	AOR (95% CI)	*p*	AOR (95% CI)
**Gender**				
Male	0.764	0.94 (0.62–1.42)	0.052	0.66 (0.44–1.01)
Female†		1		1
**Residence**				
Urban	0.001	0.40 (0.25–0.65)	0.016	1.70 (1.10–2.63)
Rural†		1		1
**Marital status**				
Married	0.707	0.82 (0.30–2.28)	0.381	1.83 (0.47–7.10)
Divorced	0.384	0.51 (0.11–2.32)	0.397	0.41 (0.05–3.27)
Widowed†		1		1
**Religion**				
Orthodox	0.93	1.04 (0.42–2.56)	0.84	1.12 (0.41–3.01)
Protestant	0.636	1.25 (0.50–3.12)	0.591	1.32 (0.48–3.65)
Other†		1		1
**Ethnicity**				
Oromo	0.205	0.46 (0.14–1.52)	0.79	1.21 (0.30–4.88)
Ahmara	0.876	0.89 (0.23–3.50)	0.816	1.20 (0.26–5.60)
Other†		1		1
**Educational status**				
Cannot read or write	0.158	0.36 (0.09–1.49)	0.005	0.20(0.07–0.61)
Can read and write	0.151	0.35 (0.09–1.46)	0.001	0.10 (0.03–0.31)
Grade 1–6	0.445	0.58 (0.15–2.33)	0.014	0.27 (0.09–0.76)
Grade 7–12	0.279	0.48 (0.13–1.82)	0.026	0.34 (0.13–0.88)
> Grade 12†		1		1
**Occupational status**				
Farmer	0.851	0.93 (0.42–2.06)	0.504	1.41 (0.52–3.83)
Housewife	0.607	1.25 (0.53–2.95)	0.055	2.71 (0.97–7.47)
Government employee	0.295	0.50 (0.14–1.82)	0.079	2.83 (0.87–9.05)
Merchant	0.9	1.07 (0.30–2.85)	0.266	1.87 (0.62–5.65)
Student	0.23	0.59 (0.25–1.40)	0.041	2.81 (1.05–7.53)
Other†		1		1
**Age**				
15–24	0.696	0.85 (0.37–1.94)	0.193	0.55 (0.22–1.35)
25–34	0.888	1.05 (0.53–2.08)	0.966	0.98 (0.46–2.12)
35–44	0.095	0.54 (0.26–1.11)	0.339	0.67 (0.30–1.52
> 45†		1		1
**Monthly household income (mean = $40.10)**				
<$40.10	0.207	1.34 (0.85–2.09)	0.106	1.45 (0.92–2.27)
>$40.10†		1		1

Reference category for outcome variables: medium.

AOR, adjusted odds ratio.

† Reference category for explanatory variables.

### Institutional aspects and visiting pattern

The likelihood ratio test (Table 2) shows the contribution of each institutional-related variable to the model. Familiarity with the provider, comfortable seating, privacy during consultation, relaying one's personal concerns related to the condition and the language of the interview contributed significantly (*p* < 0.05). The pseudo R-square value showed that the model explained about 31.1% of the variance.

**TABLE 2:** Institutional determinants of patient enablement at primary health care centres in central Ethiopia.

Explanatory variables with response options	Level of enablement (predicted)			

	Low		High	
	
	*p*	AOR (95% CI)	*p*	AOR (95% CI)
**Gender of provider**				
Male	0.313	1.22 (0.82–1.84)	0.089	0.71 (0.48–1.05)
Female†		1		1
**Familiarity with provider**				
Very good	0.116	0.36 (0.10–1.29)	0.064	2.50 (0.94–6.59)
Good	0.001	0.13 (0.00–0.45)	0.001	3.67 (1.71–7.87)
Slight	0.018	0.49 (0.27–0.89)	0.504	0.83 (0.49–1.42)
Not at all		1		1
**Number of times seen by the same provider within 12 months**				
1	0.116	0.21 (0.03–1.47)	0.415	0.43 (0.06–3.32)
2	0.148	0.24 (0.03–1.68)	0.215	0.27 (0.04–2.13)
3	0.042	0.09 (0.01–0.92)	0.032	0.08 (0.01–0.81)
≥ 4†		1		1
**Type of visit**				
Initial†		1		1
Follow-up	0.904	0.95 (0.43–2.13)	0.729	1.14 (0.54–2.39)
**Comfortable waiting area**				
Yes	0.825	1.14 (0.36–3.66)	0.842	1.14 (0.32–4.11)
No†		1		1
**Comfortable seating**				
Yes	0.012	0.47 (0.26–0.84)	0.011	3.67 (1.35–9.98)
No†		1		1
**Clean waiting area**				
Yes	0.148	0.56 (0.26–1.23)	0.663	0.78 (0.25–2.44)
No†		1		1
**Adequately private room**				
Yes	0.164	0.17 (0.01–2.08)	0.989	0.03 (0.00–0.89)
No†		1		1
**Adequately private consultation**				
Yes	0.985	0.56 (1.10–2.67)	0	0.50 (0.24–2.45)
No†		1		1
**Third party involved in discussion on patient's behalf**				
Yes	0.798	0.94 (0.61–1.47)	0.071	1.49 (0.97–2.32)
No†		1		1
**Conveying personal concerns**				
Yes†	0.001	0.36 (0.24–0.54)	0.001	2.80 (1.50–5.25)
No†		1		1
**Familiarity with interview language**				
Yes	0.03	0.31 (0.11–0.89)	0.056	0.33 (0.11–1.03)
No†				1
**Travelling time (mean = 82.4 min)**				
< 82.4 min	0.473	1.19 (0.74–1.89)	0.139	1.45 (0.89–2.39)
≥ 82.4 min†		1		1

Reference category for outcome variables: medium.

AOR, adjusted odds ratio.

† Reference category for explanatory variables.

### Perceived empathy, technical competency and non-verbal communication

Respondents’ perception of health care providers’ empathy, technical competency and non-verbal communication is shown in Table 3. Results showed that 51.3% and 51.6% of the respondents rated provider empathy and technical competency, respectively, as medium, while 53.0% rated non-verbal communication as highly favourable. Of the total number of respondents, 406 (52.9%) reported to have been told their illness, but only 287 (37.4%) reported that they were also told its cause. Only 254 (33.3%) of the respondents were given advice on how to prevent reoccurrence or development of a similar condition in the future. Close to half the respondents (*n* = 347, 45.2%) were told to return if their symptoms worsened or no improvement occurred.

**TABLE 3:** Perceived empathy, technical competency, non-verbal communication and consultation length at primary health care centres in central Ethiopia.

Variable with catergories	Frequency	%
**Perceived empathy**		
Low	182	23.7
Medium	394	51.3
High	192	25
Low	186	24.2
**Perceived technical competency**		
Medium	396	51.6
High	186	24.2
**Perceived non-verbal communication**		
Unfavourable	173	22.5
Favourable	407	53
Highly favourable	188	24.5
**Consultation length (mean = 6.26 ± 2.55 min)**		
< mean	447	62.1
≥ mean	321	37.9
**Identification of illness**		
Yes	406	52.9
No	362	47.1
**Explanation of cause of illness**		
Yes	287	37.4
No	481	62.6
**Advice on future prevention**		
Yes	254	33.3
No	514	66.7
**Return visits encouraged**		
Yes	249	98.1
No	5	1.9
Low	195	25.4
**Enablement**		
Medium	372	48.4
High	201	26.2

The results also showed that the mean duration of consultations was 6.26 ± 2.55 min (range = 2min – 20 min) and that 447 (62.1%) of the respondents reported consultation lengths below the mean value. Most of the consultations (*n* = 624, 81.3%) were shorter than patients had expected. A small percentage (*n* = 101, 13.2%) were longer than expected.

The variables shown in Table 3 were entered into a multinomial logistic regression of which the summarised output is presented in Table 4. The model's prediction accuracy was found to be 64.9%. The current model was uncertain in predicting overall patient enablement (*p* < 0.05). As shown in Table 4, perceived technical competency, non-verbal communication and empathy, advice on preventing future development of similar conditions, and encouraging follow-up visits were statistically significantly associated with patient enablement (*p* < 0.05) and contributed significantly to the model. For instance, compared to those who experienced intermediate enablement, respondents who perceived unfavourable non-verbal communication were 6.69 times more likely to feel low enablement than those who perceived highly favourable non-verbal communication (AOR = 6.69, 95% CI = 1.89–23.67, *p* = 0.003). On the other hand, explanation of the cause of the illness was significantly associated with low enablement but not with high enablement. Conversely, consultation length was significantly associated with high enablement but not with low enablement.

**TABLE 4:** Communication and perceived interaction process as determinants of patient enablement at primary health care centres in central Ethiopia.

Explanatory variables possible categories	Level of enablement (predicted)			

	Low		High	
	
	*p*	OR (95% CI)	*p*	OR (95% CI)
**Non-verbal communication**				
Unfavourable	0.003	6.69 (1.89–23.67)	0.001	4.60 (3.45–5.78)
Somewhat favourable	0.941	1.05 (0.32–3.48)	0	0.30 (0.19–0.49)
Highly favourable†		1		
**Perceived empathy**				
Low	0.004	10.88 (2.18–54.40)	0.007	0.17 (0.05–0.61)
Medium	0.08	3.99 (0.85–18.76)	0.001	0.44 (0.27–0.73)
High†		1		1
**Perceived technical competency**				
Low	0	7.26 (2.66–19.88)	0.129	0.55 (0.25–1.19)
Medium	0.024	2.96 (1.15–7.60)	0.036	0.61 (0.39–0.97)
High†		1		1
**Identification of illness**				
Yes	0.982	0.99 (0.57–1.75)	0.989	0.99 (0.57–1.75)
No†		1		1
**Explanation of cause of illness**				
Yes	0	0.26 (0.12–0.53)	0.753	0.92 (0.55–1.55)
No†		1		1
**Explanation about treatment**				
Yes	0.18	1.47 (0.84–2.56)	0.197	1.65 (0.77–3.54)
No†		1		1
**Advice on preventing similar condition**				
Yes	0.005	0.42 (0.23–0.77)	0.008	2.91 (1.32–6.43)
No†		1		1
**Return visits encouraged**				
Yes	0	0.37 (0.21–0.63)	0.022	2.03 (1.10–3.71)
No†		1		1
**Consultation length (mean = 6.26. min)**				
< 6.26 min	0.189	0.69 (0.39–1.20)	0.038	1.56 (1.03–2.39)
≥ 6.26 min†		1		1

The reference category for the outcome variable: medium.

OR, odds ratio.

† Reference category for the explanatory variables.

### Predictors of patient enablement

The fourth model was developed by entering all the variables shown to have statistically significant association (*p* < 0.05) with patient enablement in the earlier models. The summary of the predicted variable and predictors and the relative importance of each predictor are displayed in Table 5(a) and 5(b). In this model, the pseudo R-square implied that the model explained about 72% of the variance and it fitted the data adequately (*p* > 0.05). Familiarity with the providers, advice on how to prevent development of similar conditions in the future, being encouraged to return, non-verbal communication, empathy and technical competency were found to be significant predictors (*p* < 0.05) of both low and high patient enablement. However, residence and explanation of the cause of the illness were significant predictors of low patient enablement but not high enablement. Educational status, occupational status, and privacy during consultation were significantly associated with high levels of enablement.

**TABLE 5(a):** Predictors of low patient enablement at primary health care centres in central Ethiopia.

Predictors	Parameter estimates			

	*B*	*df*	*p*	AOR (95% CI)
**Residence**				
Urban	–0.85	1	0.017	0.43 (0.21–0.86)
Rural †		0		1
**Educational status**				
Cannot read or write	–0.49	1	0.606	0.61 (0.09–3.96)
Read and write	–0.72	1	0.465	0.49 (0.07–3.37)
Grade 1–6	0.29	1	0.751	1.34 (0.22–8.24)
Grade 7–12	0.41	1	0.647	1.50 (0.26–8.61)
> Grade 12†		0		1
**Occupational status**				
Farmer	–0.16	1	0.801	0.86 (0.26–2.87)
Housewife	–0.15	1	0.818	0.86 (0.24–3.06)
Government employee	0.08	1	0.932	1.08 (0.18–6.58)
Merchant	–0.53	1	0.517	0.59 (0.12–2.90)
Student	–0.88	1	0.209	0.42 (0.11–1.63)
Other†		0		1
**Familiarity with the provider**				
Very good	0.1	1	0.896	1.11 (0.23–5.29)
Good	–1.90	1	0.022	0.15 (0.03–0.76)
Slight	–0.27	1	0.431	0.77 (0.39–1.48)
Not at all†		0		1
**Comfortable seating**				
Yes	–0.19	1	0.615	0.83 (0.39–1.73)
No†		0		1
**Adequately private consultation**				
Yes	15.86	1	0.988	0.67 (0.89–2.32)
No†	15.68	1	0.988	0.45 (1.01–3.81)
**Conveying personal concerns**				
Yes	0.58	1	0.086	1.78 (0.92–3.44)
No†		0		1
**Familiarity with interview language**				
Yes	–0.62	1	0.418	0.54 (0.12–2.42)
No†		0		1
**Explanation of cause of illness**				
Yes	–1.58	1	0.001	0.21 (0.09–0.45)
No†		0		1
**Advice on preventing similar condition**				
Yes	–0.95	1	0.003	0.39 (0.21–0.73)
No†		0		1
**Return visits encouraged**				
Yes	–0.97	1	0.002	0.38 (0.21–0.69)
No†		0		1
**Non-verbal communication**				
Unfavourable	1.99	1	0.003	7.31 (1.96–27.31)
Somewhat favourable	0.07	1	0.911	1.07 (0.31–3.71)
Highly favourable†		0		1
Low	2.03	1	0.017	7.62 (1.44–40.35)
Medium	1.02	1	0.21	2.77 (0.56–13.57)
**Perceived empathy**				
High†		0		1
**Perceived technical competency**				
Low	2.06	1	0.001	7.86 (2.83–21.82)
Medium	0.79	1	0.117	2.22 (0.82–5.99)
High†		0		1
**Consultation length (mean = 6.26 min)**				
< 6.26 min	–0.23	1	0.454	0.79 (0.44–1.45)
≥ 6.26 min†		0		1

The reference category for the outcome variable: medium.

AOR, adjusted odds ratio; *B*, estimated regression co-efficient; *df*, degrees of freedom.

† Reference category for the explanatory variables.

**TABLE 5(b):** Predictors of high patient enablement at primary health care centres in central Ethiopia.

Predictors	Parameter estimates			

	*B*	*df*	*p*	AOR (95% CI)
**Residence**				
Urban	0.41	1	0.136	1.51 (0.88–2.58)
Rural†		0		1
Cannot read or write	–1.80	1	0.006	0.17 (0.05–0.59)
Read and write	–2.34	1	0.001	0.09 (0.03–0.37)
**Educational status**				
Grade 1–6	–0.83	1	0.185	0.44 (0.13–1.48)
Grade 7–12	–1.15	1	0.044	0.32 (0.10–0.97)
> Grade 12†		0		1
Farmer	0.96	1	0.118	2.60 (0.79–8.64)
**Occupational status**				
Housewife	1.86	1	0.003	6.41 (1.88–21.83)
Government employee	1.55	1	0.025	4.72 (1.21–18.34)
Merchant	1.54	1	0.024	4.68 (1.22–17.91)
Student	0.93	1	0.113	2.52 (0.80–7.91)
Other†		0		1
Very good	–0.03	1	0.961	0.98 (0.35–2.76)
**Familiarity with the provider**				
Good	0.85	1	0.036	2.34 (1.06–5.19)
Slight	–0.67	1	0.028	0.51 (0.28–0.93)
Not at all†		0		1
**Comfortable seating**				
Yes	1.05	1	0.059	2.86 (0.96–8.50)
No†		0		1
**Adequately private consultation**				
Yes	11.17	1	0.001	1.46 (3.11–5.89)
No†	12.42	0	1	
**Conveying personal concerns**				
Yes	0.13	1	0.724	1.14 (0.55–2.39)
No†		0		1
**Familiarity with interview language**				
Yes	–1.17	1	0.106	0.31 (0.06–1.28)
No†		0		1
Cause of illness explained	–0.14	1	0.565	0.87 (0.54–1.39)
		0		1
**Advice on preventing similar condition**				
Yes	1.17	1	0.005	3.21 (1.43–7.20)
No†		0		1
**Return visit encouraged**				
Yes	0.68	1	0.046	1.98 (1.01–3.87)
No†		0		1
**Non-verbal communication**				
Unfavourable	–12.46	1	0.918	3.81 (8.05–11.67)
Somewhat favourable	–1.08	1	0.001	0.34 (0.19–0.60)
Highly favourable†		0		1
**Perceived empathy**				
Low	–1.77	1	0.019	0.17 (0.04–0.75)
Medium	–0.95	1	0.001	0.39 (0.22–0.69)
High†		0		1
**Perceived technical competency**				
Low	–0.76	1	0.079	0.47 (0.20–1.09)
Medium	–0.64	1	0.018	0.53 (0.31–0.89)
High†		0		1
**Consultation length (mean = 6.26 min)**				
< 6.26 min	0.38	1	0.11	1.46 (0.91–2.35)
≥ 6.26 min†		0		1

Reference category for the outcome variable: medium.

AOR, adjusted odds ratio; *B*, estimated regression coefficient; *df*, degrees of freedom.

† Reference category for the explanatory variables.

## Discussion

The patient enablement asserts to measure patients’ ability to understand and cope with their health and illness. It indicates the quality of consultation, but without an indication of the process of the consultation. The results of this study show that consultation in primary health care is associated with a relatively low level of enablement: only 26.2% of the respondents felt that the consultation had highly enabled them. This finding is lower than findings that have been reported for other developed as well as developing countries.^19, 20^ The difference might be explained by the difference in socio-cultural and economic contexts, health services infrastructure, and health awareness and literacy. Moreover, the providers’ interpersonal skills and professional competency appear to have an impact on patient enablement. In the current study, perceived empathy, non-verbal communication and perceived technical competency were among the most important factors predicting the level of patient enablement. Other studies have also showed that empathy is crucial to the effective achievement of patient centredness and, hence, patient enablement.^8, 9, 10, 11, 18, 19, 20, 21, 22, 23^

The level of familiarity with the health care provider was also significantly associated with patient enablement in this study. Patients who experienced low enablement did not know the providers well. This finding is in line with previous findings.^24–26^ The primary health care system in Ethiopia is currently organised as part of a system for continuous health care, but with 64.6% of the respondents not being familiar with the health care provider who treated them, the situation is not reflected in the findings and may have contributed to lower enablement. Similarly, patients to whom the cause of their illness had not been explained, nor were offered advice on how to prevent similar conditions or encouraged to return for follow-up visits experienced a lower level of enablement. This is similar to a previous finding.^27^

The present study also showed that almost half of the patients (47.1%) were not told what their illness was and left the consultation without a sound and objective understanding of their illness. Moreover, 62.6% of the respondents reported that the cause of their illness had not been explained, which translates to a missed opportunity for health education. This finding is, however, inconsistent with a US study where 72% of the respondents reported that their health care providers gave them adequate information about their condition.^27, 28^ The difference may be due to the nature of the health problems, with acute infectious diseases being common in developing countries, whereas chronic conditions are more common in developed countries. However, health workers may also underestimate the importance of sharing information about the illness, thinking that patients would not be able to comprehend their explanations.

Health care providers have an ethical duty to teach the patients about disease and promotion of health, as is clearly stated in the Ethiopian medical code of ethics.^29^ However, according to the finding of this study, only 33.3% of the respondents were given advice on how to prevent the reoccurrence of the disease or how to prevent future development of similar conditions. Of those who had been given advice, 98.1% reported that they would follow the advice, which underlines the opportunity for health education and dissemination of information. To maintain continuity of care, patients should be bound to the health care system. However, in the current study more than half the patients (about 56%) were not encouraged to return for follow-up visits. This may threaten the continuity of care.

Non-verbal communication is a subtle form of communication that occurs in the initial three seconds after introduction and can continue through the entire interaction. It has as great an impact as verbal communication, but can be more easily misinterpreted.^12^ Thus, it is important for health care providers to be aware of the non-verbal messages that they convey to their patients. In the current study, non-verbal communication was a strong predictor of patient enablement. Patients who perceived non-verbal communication of the provider as unfavourable experienced lower levels of enablement. This finding is consistent with that of a systematic review of eight studies where non-verbal communication cues such as facial expression, nodding of the head, a forward-leaning posture, frequent hand gestures, open arm and leg positions and direct eye contact were positively associated with patient enablement.^30, 31^ The current study also showed that perceived technical competency was strongly associated with patient enablement. However, only 24% of the respondents reported high perceived technical competency, which resulted in the generally low enablement seen for the group.

## Conclusion

In conclusion, perceived empathy, technical competency, non-verbal communication, being told the cause of the illness, arrangement for follow-up, advice on how to prevent future development of similar conditions, familiarity with the provider and residence were found to be the main predictors of patient enablement in this study. This suggests that the parameters discussed above should be considered in medical practice. In addition, the findings can inform policy makers and health care practitioners that interpersonal interaction (including verbal and non-verbal communication), disease information and continuity of care are crucial for improving patient enablement and should seriously be considered. This study provides a basis for better prediction of factors associated with patient enablement, particularly in resource limited countries.

### Limitations of the study

The findings may be affected by the fact that facility-based studies produce more positive responses. This may result in a short-lived ‘halo effect’, with patients feeling more enabled after the consultation than later.^16^ In addition, a lack of similar studies in the region also limits the comparison of the findings.
